# Association of serum lycopene concentrations with all-cause and cardiovascular mortality among individuals with chronic kidney disease: A cohort study

**DOI:** 10.3389/fnut.2022.1048884

**Published:** 2022-12-05

**Authors:** Qiang Zhong, YongYi Piao, Shan Yin, KangYi Zhang

**Affiliations:** ^1^Department of Urology, Affiliated Hospital of North Sichuan Medical College, Nanchong, China; ^2^Department of Urology, The First Affiliated Hospital of Chongqing Medical and Pharmaceutical College, Chongqing, China; ^3^Department of Nephrology, Chongqing Public Health Medical Center, Chongqing, China; ^4^Department of Endocrinology, Chongqing Public Health Medical Center, Chongqing, China

**Keywords:** lycopene, chronic kidney disease, antioxidant, oxidative stress, mortality

## Abstract

**Background:**

Lycopene is one of the hydrocarbon carotenoids which is largely studied for its strong antioxidant and anti-inflammatory properties, as well as improvement of endothelial function and anti-arteriosclerosis effects. The use of lycopene has been shown to reduce mortality in the general population. However, few studies have examined the association between serum lycopene level and all-cause and cardiovascular mortality among participants with chronic kidney disease (CKD).

**Method:**

This study included 7,683 adults with CKD from the Third National Health and Nutrition Examination Survey (NHANES III, 1988–1994) and NHANES 2001–2006. Mortality status and cause of death were ascertained by linkage to National Death Index records through 31 December 2018. Cox proportional hazards regression models were used to estimate hazard ratios (HR) and 95% CIs for mortality from all-cause and cardiovascular disease (CVD).

**Result:**

During a median follow-up time of 309 months, there were 5,226 total deaths. The median (interquartile range) serum lycopene concentration was 20.0 (12.0, 32.0) μg/dl. After fully adjusted, restricted cubic spline analyses reported that higher serum lycopene concentrations were significantly associated with decreased risk of all-cause and CVD mortality in participants with CKD (*P* < 0.001, *P* = 0.001). When extreme quartiles of serum lycopene concentrations were compared, the multivariable-adjusted HR (95% CI) was 0.778 (0.714–0.848) for all-cause mortality (*P* < 0.001), and 0.791 (0.692–0.905) for CVD mortality (*P* < 0.001). Specifically, higher serum lycopene decreased the risk of all-cause and CVD mortality at both CKD stage 1–2 and stage 3–5. Further subgroup analyses and sensitivity analyses supported the current results.

**Conclusion:**

Higher serum lycopene was independently associated with a decreased risk of all-cause and CVD mortality in patients with CKD. These findings suggested that maintain serum lycopene concentrations could lower mortality risk in CKD patients.

## Introduction

In the USA, the incidence of chronic kidney disease (CKD) in the general population has reached nearly 15% (1, 2), and 32.2% in the elderly (3), which brings a heavy burden to individuals and society. People with CKD, have an extremely high prevalence of comorbid cardiovascular diseases (CVDs) ranging from ischemic heart disease and arrhythmias to heart failure and venous thromboembolism, and CVD is a leading cause of death (3). Chronic inflammatory state, oxidative stress (OS), and vascular endothelial dysfunction, as well as the vicious cycle formed by the three and the consequent pathological changes, are considered to be the important reasons for the high incidence rate of CVD morbidity and mortality in CKD patients (4). Among them, OS holds a key position as a central link of the intricated pathways involved in the progression of CKD (5–7). Therefore, antioxidant therapy has become one of the important measures to prevent and delay CKD progression.

Lycopene is a carotenoid (C40H56) present in red fruit and vegetables, including tomato, papaya, red pepper, watermelons, among others (8). Dietary Lycopene absorption is influenced by several factors, including release from the food matrix, cooking temperatures, and the presence of oil and other lipid-soluble compounds (9). Lycopene is transported from the intestinal mucosa to the general circulation *via* the lymphatic system (10). In the blood, lycopene is mainly transported by low-density lipoproteins (LDL) (11). OS as well as hyperactivity of the endogenous enzymes responsible for generation of reactive oxygen species (ROS) and nitric oxide (NO) may deplete lycopene reserves in human cells and tissues (12). CKD patients are accompanied by long-lasting OS, which may cause lycopene depletion faster than normal population. Clinical research shown that serum lycopene concentrations in hemodialysis patients were lower when compared with healthy controls (13, 14).

Experimental evidence has been presented that the use of lycopene can inhibit NF-κB activation, increase superoxide dismutase activity, improve the NO bioavailability and protect vascular endothelial function, which subsequently improve OS, reduce chronic inflammation (8, 15–17). Also, lycopene can inhibit LDL-cholesterol peroxidation, which can directly damage the kidney and increase the risk of atherosclerosis and CVD (18–21). Further, evidence from epidemiological studies revealed that higher lycopene concentrations were associated with lower risk of ischemic stroke and CVD (22, 23). Hence, higher lycopene concentrations may benefit patients with CKD.

In fact, higher serum lycopene concentrations have been reported to be associated with lower risk of CKD in the general population (24) and reduce atherogenesis in patients receiving hemodialysis (25). However, the relationship between serum lycopene concentrations and mortality has not been elucidated yet in patients with CKD. To fill these knowledge gaps, this study focused on the association serum lycopene concentration and all-cause and CVD mortality, and the individuals come from the National Health and Nutrition Examination Survey (NHANES).

## Materials and methods

### Population resource

Generally reported, NHANES assessed the health and nutritional status of the USA civilian, non-institutionalized population (26). The present study was a prospective cohort study enrolling individuals from NHANES III (1988–1994) and 2001–2006 with lycopene measures and assessment of CKD.

### Assessment of lycopene levels and chronic kidney diseases

As reported, serum lycopene levels were measured using high-performance liquid chromatography (HPLC). As previously reported (27), HPLC relies on a pump to push a pressurized liquid solvent containing a sample mixture through a column packed with solid adsorbent material. Each component in the sample interacts with the sorbent material slightly differently, resulting in different flow rates for the different components and separation of the components as they exit the column. CKD was defied as an estimated glomerular filtration rate (eGFR) and urinary albumin-to-creatinine ratio based on kidney diseases improving global outcomes (KDIGO) guideline (28). The chronic kidney disease epidemiology collaboration equation (CKD-EPI) was used to calculate eGFR. CKD was graded as follows: Stage 1, eGFR ≥ 90 ml/min/1.73 m^2^ with albuminuria; stage 2, eGFR of 60–89 ml/min/1.73 m^2^ and albuminuria; stage 3, eGFR of 30–59 ml/min/1.73 m^2^; stage 4, eGFR of 15–29 ml/min/1.73 m^2^; and stage 5, eGFR < 15 ml/min/1.73 m^2^.

### Mortality outcome ascertainment

Mortality information was obtained by linking to the National Death Index through 31 December 2018. The follow-up duration was calculated from the time participating in this survey to the date of death or 31 December 2018. In this database, the International Statistical Classification of Diseases and Related Health Problems, Tenth Revision (ICD-10) was used to identify the underlying cause of death. In our current study, we evaluated the impact of lycopene levels on both all-cause and CVD mortality. Specifically, cardiovascular death was identified by codes I00–I09, I11, I13, and I20–I51.

### Covariates assessment

We extracted demographic characteristics, laboratory tests, and chronic comorbidity data from NHANES III and NHANES 2001–2006. Demographic characteristics included age, sex, body mass index (BMI), race/ethnicity, education levels, family poverty-to-income ratio, smoking status, drinking status, anti-diabetic drug use, and anti-hypertensive drug use. Laboratory data included serum triglycerides, total cholesterol, and uric acid. Chronic co-morbidity included diabetes and hypertension. Education levels were classified into less than high school, high school, and college or above. Family income-to-poverty ratio was calculated by dividing total family income by the poverty threshold. Smoking status was assessed and were classified into current smoker, former smoker (not smoking now but > 100 cigarettes in life), and never smoker (< 100 cigarettes in life). Drinking status were grouped into current heavy use (≥ 3 drinks/day for males and ≥ 4 for females), moderate use (≥ 2 drinks/day for males and ≥ 3 for females), mild use (current use but < 2 drinks/day for males and < 3 for females), and never. Diabetes was diagnosed based on the question “Are you told to have diabetes by a doctor,” the use of antidiabetic drug use, or laboratory tests, including HbA1c ≥ 6.5%, fast glucose ≥ 7.1 mmol/L, random glucose ≥ 11.1 mmol/L, or 2-h glucose ≥ 11.1 mmol/L after oral glucose tolerance tests.

### Missing data imputation

Missing data included income to poverty ratio (*n* = 733/7,683, 9.5%), BMI (*n* = 1,093/7,683; 14.2%), triglycerides (*n* = 12/7,683, < 0.1%), uric acid (*n* = 40/4,322, < 0.1%), and total cholesterol (*n* = 1,826/7,683, 23.7%). Multiple imputation with chained equations were used to imputed these missing values, where linear regression was used to impute normally distributed continuous variables, predictive mean matching was used for non-normally distributed continuous variables, and logistic regression for binary variables.

### Statistical analyses

Lycopene levels were categorized into four categories based on the quartile. Continuous variables are expressed as mean (standard deviation) and categorical variables are presented as numbers (proportions). Continuous and categorical demographic variables were compared using analysis of variance (ANOVA) and Chi-square tests, respectively.

We first conducted restricted cubic spline analyses to evaluate the association between serum lycopene as the continuous variable between all-cause and CVD mortality. Then, Cox proportional hazards analyses were conducted to identify the independent effect of different quartiles of lycopene levels on all-cause and cardiovascular-cause mortality. In Model 1, the estimates were adjusted for age and sex. Model 2 was further adjusted for race/ethnicity, education, income-to-poverty ratio, BMI, triglycerides, uric acid, and total cholesterol based on model 1. Model 3 was the fully adjusted model, further adjusted for smoking status, drinking status, diabetes, anti-diabetic drug use, hypertension, and anti-hypertensive drug use based on model 2. In the Model 4, lycopene levels were analyzed as the continuous variable adjusted by these variables in the model 3. We further applied stratification analysis for associations of lycopene levels with all-cause and cardiovascular-cause mortality based on age (< 65 or ≥ 65 years), sex, race/ethnicity (non-Hispanic white or other), BMI (< 30 or ≥ 30 kg/m^2^), serum triglycerides (≥ 200 mg/dl, or < 200 mg/dl), serum total cholesterol (≥ 240 mg/dl, or < 240 mg/dl), diabetes (Yes or No), hypertension (Yes or No), current smoking status (Yes or No), current drinking status (Yes or No), and CKD stage (stage 1–2, or stage 3–5). Sensitivity analyses were performed by: (1) excluding the participants who died within 12 months; (2) excluding the participants with missing data.

## Results

**FIGURE 1:**
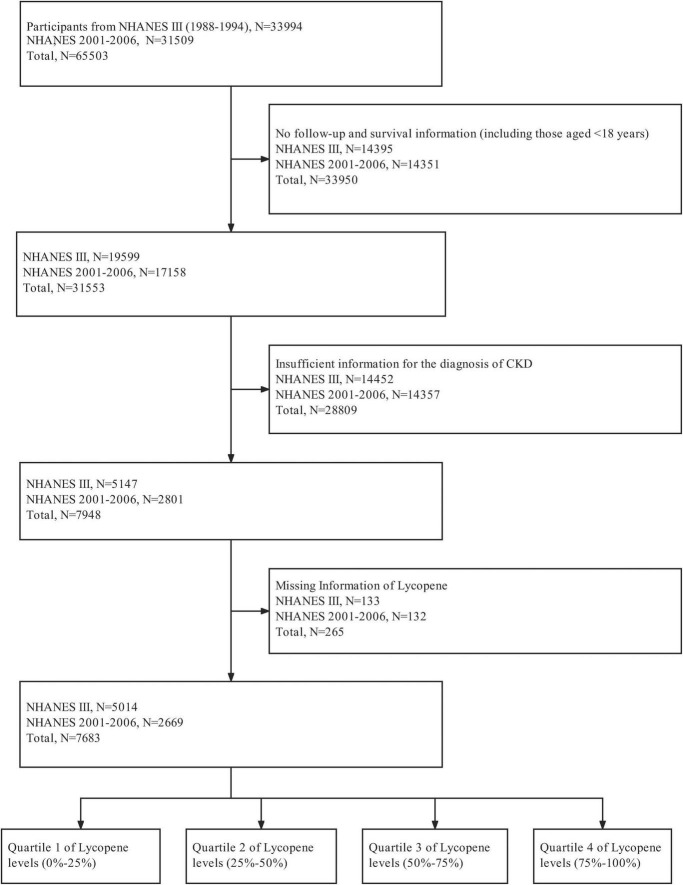
Flow chart.

The study population included 7,683 participants and the screening process was shown in Figure 1. The baseline characteristics according to quartiles of serum lycopene concentrations were shown in Table 1. The mean age of the subjects was 63.1 ± 18.5 years, and 42% were male. Non-Hispanic Whites accounted for 53 and 54% of the participants were at the educational level of high school or higher. Among these participants, the median (interquartile range) serum lycopene concentration was 20.0 (12.0, 32.0) μg/dl. Participants with higher serum lycopene concentrations were more likely to be younger, non-Hispanic white, non-alcohol drinker, never smoker, have higher education level, higher family income, higher value of eGFR.

**TABLE 1:** Baseline characteristics of patients with chronic kidney disease in NHANES III and NHANES 2001–2006.

	Serum lycopene (μg/dl)					
	
Characteristics	Total	Quartile 1	Quartile 2	Quartile 3	Quartile 4	*P*-value
Patients, *n*	7,683	1,970	1,881	1,911	1,921	
Age, years	63.1 (18.5)	70.1 (14.6)	64.6 (17.6)	60.1 (18.8)	57.5 (20.0)	< 0.001
Male, sex (%)	3,225 (42.0%)	852 (26.4%)	702 (21.8%)	818 (25.4%)	853 (26.4%)	
**Race (%)**						0.006
Mexican American	1,251 (16.3%)	301 (15.3%)	300 (15.9%)	338 (17.7%)	312 (16.2%)	
Non-Hispanic Black	2,044 (26.6%)	556 (28.2%)	482 (25.6%)	515 (26.9%)	491 (25.6%)	
Non-Hispanic White	4,049 (52.7%)	1,033 (52.4%)	1,034 (55.0%)	976 (51.1%)	1,006 (52.4%)	
Other	339 (4.4%)	80 (4.1%)	65 (3.5%)	82 (4.3%)	112 (5.8%)	
**Education (%)**						< 0.001
College or above	1,517 (19.7%)	270 (13.7%)	288 (15.3%)	387 (20.3%)	572 (29.8%)	
High school	2,601 (33.9%)	622 (31.6%)	643 (34.2%)	614 (32.1%)	722 (37.6%)	
Less than high school	3,565 (46.4%)	1,078 (54.7%)	950 (50.5%)	910 (47.6%)	627 (32.6%)	
Poverty to income ratio	2.4 (1.7)	2.0 (1.5)	2.4 (1.7)	2.5 (1.7)	2.5 (1.7)	< 0.001
BMI, kg/m^2^	28.0 (6.0)	27.4 (5.9)	27.7 (5.8)	28.4 (5.9)	28.5 (6.2)	< 0.001
eGFR, ml/min/1.73 m^2^	61.0 (23.0)	54.5 (17.5)	57.5 (18.8)	62.2 (22.6)	70.0 (28.5)	< 0.001
Serum triglycerides, mg/dl	163.4 (133.9)	157.2 (99.8)	158.6 (102.1)	162.9 (124.8)	175.0 (188.9)	< 0.001
Serum uric acid, mg/dl	5.9 (1.7)	5.9 (1.7)	5.8 (1.6)	5.8 (1.6)	5.9 (1.6)	0.086
Serum total cholesterol, mg/dl	250.2 (112.0)	223.0 (86.5)	235.2 (94.3)	251.4 (109.7)	291.6 (138.3)	< 0.001
Urine albumin, μg/ml	158.8 (645.9)	138.7 (649.7)	147.4 (712.9)	163.0 (582.7)	186.5 (632.2)	0.106
Urine creatinine, μmol/l	3,402.4 (6,252.8)	1,256.0 (3,834.8)	1,787.6 (4,177.0)	3,469.7 (6,228.2)	7,117.7 (8,036.0)	< 0.001
Urinary ACR, mg/g	8,498.8 (50,544.2)	10,599.8 (54,146.9)	9,485.5 (57,068.8)	8,190.3 (45,181.0)	5,684.9 (44,515.3)	0.017
**CKD (%)**						< 0.001
Stage 1–2	2,396 (31.2%)	411 (20.9%)	464 (24.7%)	651 (34.1%)	870 (45.3%)	
Stage 3–5	5,287 (68.8%)	1,559 (79.1%)	1,417 (75.3%)	1,260 (65.9%)	1,051 (54.7%)	
Serum lycopene [median (IQR)], μg/dl	20.0 (12.0, 32.0)	8.0 (6.0, 10.0)	16.0 (14.0, 18.0)	25.0 (23.0, 28.0)	42.0 (36.0, 51.3)	
Serum alpha-carotene [median (IQR)], μg/dl	3.6 (1.8, 6.0)	3.0 (1.0, 5.0)	4.0 (2.0, 6.0)	4.0 (2.0, 6.0)	4.0 (2.0, 6.4)	< 0.001
Serum beta-carotene [median (IQR)], μg/dl	16.9 (9.2, 28.2)	14.0 (8.0, 24.0)	16.0 (9.0, 28.0)	18.0 (10.0, 30.0)	19.0 (10.7, 31.4)	< 0.001
Serum beta-cryptoxanthin [median (IQR)], μg/dl	8.0 (5.0, 12.2)	6.0 (4.0, 10.0)	7.7 (5.0, 12.0)	8.9 (6.0, 13.4)	9.3 (6.0, 14.2)	< 0.001
Serum lutein [median (IQR)], μg/dl	19.0 (13.0, 27.0)	17.4 (12.0, 25.0)	19.0 (13.9, 27.0)	20.0 (14.0, 29.0)	19.0 (13.2, 27.0)	< 0.001
**Diabetes mellitus (%)**						0.001
No	6,034 (78.5%)	1,552 (86.7%)	1,503 (79.9%)	1,532 (80.2%)	1,447 (75.3%)	
Yes	1,649 (21.5%)	418 (23.3%)	378 (20.1%)	379 (19.8%)	474 (24.7%)	
**Hypertension (%)**						0.032
No	3,574 (46.5%)	932 (47.3%)	901 (47.9%)	903 (47.3%)	838 (43.6%)	
Yes	4,109 (53.5%)	1,038 (52.7%)	980 (52.1%)	1,008 (52.7%)	1,083 (56.4%)	
**Smoking status (%)**						0.002
Current	1,154 (15.0%)	310 (15.7%)	251 (13.3%)	304 (15.9%)	289 (15.0%)	
Former	2,642 (34.4%)	728 (37.0%)	662 (35.2%)	641 (33.5%)	611 (31.8%)	
Never	3,887 (50.6%)	932 (47.3%)	968 (51.5%)	966 (50.6%)	1,021 (53.2%)	
**Drinking status (%)**						0.073
Heavy	609 (7.9%)	151 (7.7%)	130 (6.9%)	158 (8.3%)	170 (8.8%)	
Mild	2,115 (27.5%)	513 (26.0%)	516 (27.4%)	532 (27.8%)	554 (28.8%)	
Moderate	633 (8.2%)	144 (7.3%)	163 (8.7%)	166 (8.7%)	160 (8.3%)	
Never	4,326 (56.3%)	1,162 (59.0%)	1,072 (57.0%)	1,055 (55.2%)	1,037 (54.0%)	
**Diabetes medicine use (%)**						< 0.001
No	6,779 (88.2%)	1,769 (89.8%)	1,675 (89.0%)	1,701 (89.0%)	1,634 (85.1%)	
Yes	904 (11.8%)	201 (10.2%)	206 (11.0%)	210 (11.0%)	287 (14.9%)	
**Hypertension medicine use (%)**						< 0.001
No	5,399 (70.3%)	1,183 (60.0%)	1,260 (67.0%)	1,376 (72.0%)	1,580 (82.2%)	
Yes	2,284 (29.7%)	787 (40.0%)	621 (33.0%)	535 (28.0%)	341 (17.8%)	

Normally distributed continuous variables are described as means ± SD, and continuous variables without a normal distribution are described as medians (interquartile ranges). Categorical variables are presented as numbers (percentages). IQR, interquartile range; BMI, body mass index; eGFR, estimated glomerular filtration rate; ACR, albumin-creatinine ratio; CKD, chronic kidney disease.

**FIGURE 2:**
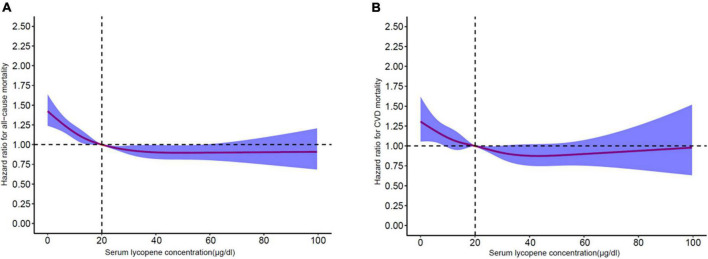
**(A)** Restricted cubic spline analyses between serum lycopene concentrations and all-cause mortality. **(B)** Restricted cubic spline analyses between serum lycopene concentrations and cardiovascular-cause mortality.

Of 7,683 CKD participants, there were 5,226 deaths during a median follow-up time of 309 months. After multivariable adjustment, higher serum lycopene concentrations were significantly associated with the reduction of all-cause mortality in participants with CKD (Table 2). The multivariable adjusted hazard ratios (HRs) (95% CI) across quartiles of serum lycopene concentrations from low to high were 1 (reference), 0.903 (0.84–0.971), 0.791 (0.732–0.854), and 0.778 (0.714–0.848) (*P*_*trend*_ < 0.001). In model 4, for every 1% increase in serum concentration, all-cause mortality decreased by 0.7% (HR 0.993, 95% CI 0.991–0.995, *P* < 0.001). Restricted cubic spline analyses showed similar results between serum lycopene concentration and all-cause mortality (*P* < 0.001) (Figure 2A). Like associations seen with all-cause mortality, higher serum lycopene concentrations were associated with decreased CVD mortality (Table 3). The multivariable adjusted HRs (95% CI) across quartiles of serum lycopene concentrations were 1 (reference), 0.91 (0.813–1.018), 0.818 (0.726–0.923), and 0.791 (0.692–0.905) (*P*_*trend*_ < 0.001). For every 1% increase in serum concentration, the risk of CVD mortality decreased by 0.6% (HR 0.994, 95% CI 0.991–0.998, *P* < 0.001). A non-linear dose-response relationship of serum lycopene concentration and CVD mortality was also demonstrated (*P* = 0.001) (Figure 2B).

**TABLE 2:** All-cause mortality according to quartiles of serum lycopene concentrations among patients with CKD.

	Quartiles of serum lycopene level (μg/dl)				
	
	Quartiles 1	Quartiles 2	Quartiles 3	Quartiles 4	*P*_*trend*_
Median (IQR)	8.0 (6.0, 10.0)	16.0 (14.0, 18.0)	25.0 (23.0, 28.0)	42.0 (36.0, 51.3)	
Rang	0–12.0	12.0–20.0	20.1–31.9	32.00–151.0	
Death (%)	1,685 (86)	1,394 (74)	1,150 (60)	997 (52)	
Model 1	Reference	0.909 (0.847–0.977)	0.794 (0.736–0.857)	0.826 (0.763–0.894)	< 0.001
Model 2	Reference	0.908 (0.845–0.976)	0.795 (0.736–0.859)	0.805 (0.74–0.875)	< 0.001
Model 3	Reference	0.903 (0.84–0.971)	0.791 (0.732–0.854)	0.778 (0.714–0.848)	< 0.001
Model 4	0.993 (0.991–0.995)				< 0.001

Data are presented as HR (95% CI) unless indicated otherwise. Model 1: Adjusted for age (continuous) and sex (male or female); Model 2: Model 1 plus race/ethnicity, education level, poverty to income ratio, BMI, uric acid, triglycerides, total cholesterol; Model 3: Model 2 plus smoking status, drinking status, diabetes, hypertension, diabetes medicine, hypertension medicine; Model 4: continues model (each per 1% increase in serum concentrations), adjusted by variables in model 3.

**TABLE 3:** Cardiovascular disease mortality according to quartiles of serum lycopene concentrations among patients with CKD.

	Quartiles of serum lycopene level (μg/dl)				
	
	Quartiles 1	Quartiles 2	Quartiles 3	Quartiles 4	*P*_*trend*_
Median (IQR)	8.0 (6.0, 10.0)	16.0 (14.0, 18.0)	25.0 (23.0, 28.0)	42.0 (36.0, 51.3)	
Rang	0–12.0	12.0–20.0	20.1–31.9	32.00–151.0	
Death (%)	716 (1,970)	571 (1,881)	473 (1,911)	395 (1,921)	
Model 1	Reference	0.905 (0.81–1.011)	0.808 (0.718–0.908)	0.806 (0.712–0.913)	< 0.001
Model 2	Reference	0.903 (0.808–1.01)	0.806 (0.716–0.909)	0.784 (0.688–0.893)	< 0.001
Model 3	Reference	0.91 (0.813–1.018)	0.818 (0.726–0.923)	0.791 (0.692–0.905)	< 0.001
Model 4	0.994 (0.991–0.998)				< 0.001

Data are presented as HR (95% CI) unless indicated otherwise. Model 1: Adjusted for age (continuous) and sex (male or female); Model 2: Model 1 plus race/ethnicity, education level, poverty to income ratio, BMI, uric acid, triglycerides, total cholesterol; Model 3: Model 2 plus smoking status, drinking status, diabetes, hypertension, diabetes medicine, hypertension medicine; Model 4: continues model (each per 1% increase in serum concentrations), adjusted by variables in model 3.

**FIGURE 3:**
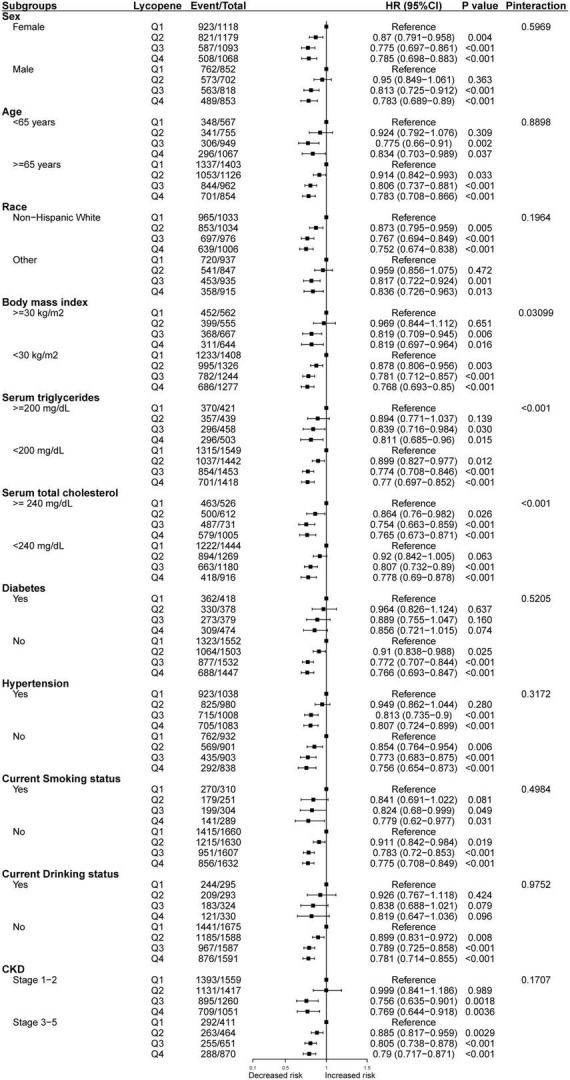
Subgroup analyses of the association between serum lycopene concentrations and all-cause mortality.

**FIGURE 4:**
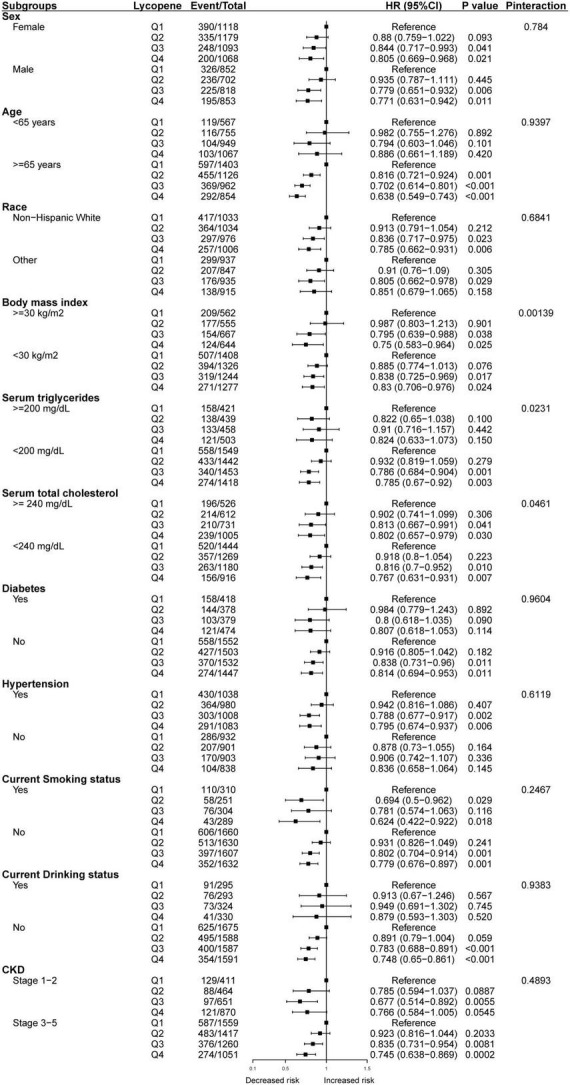
Subgroup analyses of the association between serum lycopene concentrations and cardiovascular-cause mortality.

Consistent results were observed between serum lycopene concentrations and all-cause mortality when analyses were stratified by sex, age, race, BMI, serum triglycerides, serum total cholesterol, diabetes, hypertension, current smoking status, current drinking status, and CKD stage (Figure 3). We also conducted subgroup analyses of the association between serum lycopene concentrations and CVD mortality (Figure 4). The relationship seems to be more obvious and significant in participant who were older, non-Hispanic White, non-smoker, non-drinker and without hyperlipidemia, hypercholesterolemia, or diabetes.

The results also showed that with increasing serum lycopene concentration, all-cause and CVD mortality decreased progressively in participants with CKD stage 3–5. The subgroup analyses were also conducted among these patients, and the result remained consistent with the main analysis (Supplementary Tables 1, 2). In particular, the upper quartile serum concentrations of lycopene were associated with a statistically significant reduction in all-cause and CVD mortality in CKD stage 3–5 patients with diabetes.

In sensitive analyses, the same analyses (using model 3) were repeated after excluding people died within the first follow-up 12 months or using data before multiple imputation (Supplementary Table 3). The inverse association of serum lycopene concentration with all-cause and CVD mortality were not changed.

## Discussion

In this study, we examined the association between serum lycopene concentrations and all-cause and CVD mortality among participants with CKD. We found that higher serum lycopene was independently associated with a decreased risk of all-cause and CVD mortality after adjusting for major known risk factors such as age, sex, education, serum triglycerides, total cholesterol, smoking/drinking status, diabetes, hypertension. A variety of subgroup analyses and sensitivity analyses confirmed the robustness of overall findings.

Oxidative stress is defined as a state of imbalance between excess oxidant (free) radicals and insufficient degradation of these radicals by the antioxidant system (29). There is substantial evidence to suggest that CKD patients are characterized by enhanced OS, even in early stages (30, 31). In the presence of renal dysfunction, high ROS production and decreased clearance of pro-oxidant substances, together with damage to the antioxidant system, are responsible for the pro-oxidant environment in patients with CKD (30–32). Furthermore, endothelial dysfunction, pro-inflammation status and CVD, the major cause of death in patients with CKD, are also linked to increased levels of OS (29).

Lycopene is a lipid soluble compound with 40 carbon atoms and contains 13 linearly arranged double bonds, 11 of which are conjugated (8). The elongated carbon chain with conjugated double bonds makes lycopene the most potent single oxygen and free radical scavenger among carotenoids (33, 34). The main protective effect of lycopene is achieved through the inactivation of ROS and the extinction of free radicals (35). In addition to its antioxidant capacity, lycopene also exhibits anti-inflammatory, anti-atherosclerosis properties and improved endothelial function (36). Lycopene can attach to LDL cholesterol in plasma and provide protection against atherosclerosis *via* lipid peroxidation (36). As a powerful antioxidant, there has been a growing body of evidence supporting the direct role in decreasing the all-cause mortality and preventing the occurrence and progression of CVD. Li and colleague’s umbrella review showed that dietary lycopene intake or serum lycopene was inversely associated with all-cause mortality, prostate cancer, stroke, CVD, and metabolic syndrome (37). In another comprehensive meta-analysis (38), the results showed that high-intakes or high-serum concentration of lycopene are associated with significant reductions in the risk of stroke (26%), mortality (37%), and CVDs (14%). Shardell et al. suggested that lycopene was the carotenoid most strongly predictive of all-cause mortality in the general population (39).

In this study, we founded that the concentration of lycopene tended to decline with increasing age in CKD patients. This is consistent with the study by Semba et al. who confirmed that the serum lycopene concentration of older participants was statistically lower than that of young after matching with similar ethnic and dietary backgrounds (40). On the other hand, our results showed that higher concentrations of lycopene can significantly reduce risk of all-cause mortality and CVD mortality in older (≥ 65 years) CKD patients, but not in patients younger than 65 years. Obesity is an independent risk-factor in many chronic diseases and may increase OS (41, 42). Levels of OS are correspondingly higher in obese CKD patients than in non-obese CKD patients. Our results showed that higher concentrations of lycopene also significantly reduced risk of all-cause mortality and CVD mortality in obese CKD patients. For patients with CKD stage 1–5 and diabetes, pooled results showed that higher lycopene concentrations were associated with a reduction in all-cause mortality and CVD mortality, but the difference was not statistically significant; however, in CKD stages 3–5 patients with diabetes, this difference was statistically significant. It can be seen from the above that there were a clear and significant health benefits of increasing lycopene concentrations in the CKD patients that may have higher levels of OS.

The antioxidant capacity of carotenoids is well known, but at high concentrations and under unusual conditions such as high intracellular OS, high oxygen tension, and low levels of endogenous antioxidants, carotenoids can function as pro-oxidants (43–45). Clinical trial has shown that high does β-carotene supplementation may increase lung cancer incidence in male smokers (46). Higher concentrations of serum β-carotene were significantly associated with an increased risk of cardiovascular mortality among patients with diabetes (47). For lycopene, there is no epidemiological evidence that lycopene in different concentrations exhibits the conversion between anti-oxidation and pro-oxidation properties, or harmful effects at high concentrations (48). Our results also showed that high concentrations of serum lycopene can significantly reduce all-cause mortality and CVD mortality even in patients with CKD stage 3–5 and diabetes, who are at a high level of OS and pro-inflammatory status.

There is no official recommended for the daily intake of lycopene. Tomatoes can provide almost 85% of the lycopene (49), and heat-processed tomato products can micronize lycopene and promote its intestinal absorption (8, 50). However, the absorption and bio-availability of lycopene from dietary sources is extremely low, and a large proportion of dietary lycopene is excreted from the body in undigested form (14). Furthermore, the accumulation of lycopene varies in tissues, and this accumulation is influenced by genetic factors and phenotypic (51, 52); this results in individual differences in the bioavailability and distribution of lycopene. Due to the poor statistical correlation between diet and serum lycopene levels (14), circulating levels of lycopene concentration were superior to self-reported measures of dietary intake in assessing the relationship between lycopene and chronic disease (53, 54). Previous studies showed that an intake of 5–7 mg of lycopene per day was recommended for healthy people (55), and higher doses of lycopene (35–75 mg/day) may be required in the setting of cancer or CVD (56).

This study has some limitations. First, although this was a prospective follow-up study, the number of follow-ups visit for every participant was extremely limited; Second, dietary habits recorded at baseline may significantly change during follow-up, especially when the patient’s eGFR decrease progressively or develop intercurrent disorders. Third, the lycopene concentration obtained at the time of testing may not be representative of the patient’s long-term dietary patterns and the actual condition. Fourth, socioeconomic situations may change, and alterations in exercise and other environmental factors like medication use may affect the development of morbidity and death; Fifth, these results are based on USA adults with CKD, which may limit the generalizability to other populations; Finally, the possibility of residual and unknown confounding cannot be eliminated.

## Conclusion

Higher serum lycopene was independently associated with a decreased risk of all-cause and CVD mortality in patients with CKD. These findings suggested that maintain serum lycopene concentrations could lower mortality risk in CKD patients.

## Data availability statement

The original contributions presented in this study are included in the article/Supplementary material, further inquiries can be directed to the corresponding author.

## Ethics statement

This study involved secondary data analysis of a nationally representative publicly available dataset. The study we conducted was exempt from institutional review for this reason. Written informed consent for participation was not required for this study in accordance with the national legislation and the institutional requirements.

## Author contributions

QZ and YP analyzed the data and drafted the manuscript. SY provided technical guidance. KZ designed the study and revised the manuscript. All authors approved the final version of the manuscript, ensure the accuracy and integrity of the work, and agree to be accountable for all aspects of the work.
