# Supplementary material for: Association of serum lycopene concentrations with all-cause and cardiovascular mortality among individuals with chronic kidney disease: A cohort study
Source: Front Nutr. 2022 Dec 5;9:1048884. doi: 10.3389/fnut.2022.1048884 (PMC9760801; doi:10.3389/fnut.2022.1048884)
Supplement: Supplementary file 1 [file Table_1.docx]

**Supplementary Table 1 Subgroup analyses of the associations between serum lycopene concentrations and all-cause mortality among patient with CKD stage 3-5**

|  | **Serum lycopene concentration(μg/dl)** | | | | | | | | | | |  |
| --- | --- | --- | --- | --- | --- | --- | --- | --- | --- | --- | --- | --- |
| **Characteristics** | **Quartile 1** | | **Quartile 2** | | **P** | **Quartile 3** | | **p** | **Quartile 4** | | **p** | **P _interaction_** |
|  | **No. deaths/total** | **Reference** | **No. deaths/total** | **HR (95%CI)** |  | **No. deaths/total** | **HR (95%CI)** |  | **No. deaths/total** | **HR (95%CI)** |  |  |
| Sex |  |  |  |  |  |  |  |  |  |  |  | 0.613 |
| female | 794/911 | 1 | 696/900 | 0.858 (0.773-0.952) | 0.0039 | 486/727 | 0.8 (0.713-0.898) | 0.0002 | 388/604 | 0.776 (0.68-0.886) | 0.0002 |  |
| male | 599/648 | 1 | 435/517 | 0.932 (0.82-1.059) | 0.2792 | 409/533 | 0.811 (0.709-0.926) | 0.002 | 321/447 | 0.807 (0.693-0.941) | 0.0061 |  |
| Age, year |  |  |  |  |  |  |  |  |  |  |  | 0.474 |
| ＜65 | 223/340 | 1 | 224/447 | 0.827 (0.683-1.001) | 0.0506 | 191/470 | 0.626 (0.513-0.765) | <0.001 | 166/399 | 0.686 (0.552-0.852) | 0.0007 |  |
| ≥65 | 1170/1219 | 1 | 907/970 | 0.792 (0.725-0.866) | <0.001 | 704/790 | 0.686 (0.623-0.755) | <0.001 | 543/652 | 0.618 (0.553-0.69) | <0.001 |  |
| Race/ethnicity |  |  |  |  |  |  |  |  |  |  |  | 0.124 |
| Non-Hispanic White | 835/888 | 1 | 750/863 | 0.915 (0.827-1.012) | 0.0852 | 573/741 | 0.8 (0.717-0.893) | 0.0001 | 487/667 | 0.775 (0.685-0.876) | <0.001 |  |
| Other | 558/671 | 1 | 381/554 | 0.861 (0.753-0.984) | 0.0275 | 322/519 | 0.81 (0.702-0.935) | 0.0039 | 222/384 | 0.836 (0.705-0.992) | 0.0404 |  |
| BMI, kg/m^2^ |  |  |  |  |  |  |  |  |  |  |  | 0.034 |
| ≥30 | 361/423 | 1 | 305/392 | 0.944 (0.807-1.105) | 0.474 | 274/399 | 0.837 (0.711-0.986) | 0.0333 | 213/333 | 0.809 (0.669-0.977) | 0.0281 |  |
| ＜30 | 1032/1136 | 1 | 826/1025 | 0.863 (0.786-0.948) | 0.0022 | 621/861 | 0.791 (0.714-0.877) | <0.001 | 496/718 | 0.777 (0.691-0.874) | <0.001 |  |
| Serum triglycerides, mg/dl |  |  |  |  |  |  |  |  |  |  |  | 0.529 |
| ≥200 | 313/337 | 1 | 294/348 | 0.911 (0.773-1.073) | 0.2637 | 224/297 | 0.865 (0.724-1.035) | 0.1129 | 212/281 | 0.877 (0.724-1.063) | 0.182 |  |
| ＜200 | 1080/1222 | 1 | 837/1069 | 0.873 (0.796-0.958) | 0.004 | 671/963 | 0.785 (0.71-0.867) | <0.001 | 497/770 | 0.761 (0.678-0.855) | <0.001 |  |
| Serum total cholesterol, mg/dl |  |  |  |  |  |  |  |  |  |  |  | 0.414 |
| ≥240 | 390/423 | 1 | 403/468 | 0.807 (0.7-0.93) | 0.0031 | 364/472 | 0.719 (0.621-0.832) | <0.001 | 409/549 | 0.722 (0.623-0.837) | <0.001 |  |
| ＜240 | 1003/1136 | 1 | 728/949 | 0.923 (0.837-1.018) | 0.1086 | 531/788 | 0.839 (0.752-0.936) | 0.0016 | 300/502 | 0.816 (0.711-0.938) | 0.0041 |  |
| Diabetes |  |  |  |  |  |  |  |  |  |  |  | 0.042 |
| yes | 274/293 | 1 | 238/262 | 0.808 (0.675-0.967) | 0.0198 | 183/214 | 0.851 (0.7-1.033) | 0.1026 | 182/228 | 0.739 (0.599-0.911) | 0.0047 |  |
| No | 1119/1266 | 1 | 893/1155 | 0.922 (0.704-1.207) | 0.1219 | 712/1046 | 0.794 (0.72-0.875) | <0.001 | 527/823 | 0.807 (0.72-0.904) | <0.001 |  |
| Hypertension |  |  |  |  |  |  |  |  |  |  |  |  |
| yes | 775/847 | 1 | 684/783 | 0.937 (0.843-1.041) | 0.2258 | 546/680 | 0.829 (0.741-0.928) | 0.0011 | 493/660 | 0.788 (0.696-0.893) | 0.0002 |  |
| no | 618/712 | 1 | 447/634 | 0.826 (0.729-0.937) | 0.003 | 349/580 | 0.775 (0.674-0.89) | 0.0003 | 216/391 | 0.812 (0.687-0.959) | 0.0144 |  |
| Current smoking status |  |  |  |  |  |  |  |  |  |  |  | 0.386 |
| yes | 220/238 | 1 | 136/174 | 0.817 (0.653-1.022) | 0.0773 | 143/179 | 0.832 (0.665-1.041) | 0.1078 | 84/117 | 0.774 (0.577-1.039) | 0.0878 |  |
| no | 1173/1321 | 1 | 995/1243 | 0.892 (0.818-0.973) | 0.0096 | 752/1081 | 0.796 (0.724-0.874) | <0.001 | 625/934 | 0.775 (0.698-0.860) | <0.001 |  |
| Current drinking status |  |  |  |  |  |  |  |  |  |  |  | 0.949 |
| yes | 201/228 | 1 | 160/198 | 0.877 (0.709-1.085) | 0.2278 | 129/183 | 0.817 (0.652-1.024) | 0.079 | 77/128 | 0.925 (0.7-1.221) | 0.5808 |  |
| no | 1192/1331 | 1 | 971/1219 | 0.878 (0.805-0.958) | 0.0034 | 766/1077 | 0.802 (0.73-0.881) | <0.001 | 632/923 | 0.771 (0.695-0.856) | <0.001 |  |

Data are presented as HR (95% CI). Adjusted for age (continuous) ，sex (male or female)，race/ethnicity, education level, poverty to income ratio, BMI, uric acid, triglycerides, total cholesterol, smoking status,

drinking status, diabetes, hypertension, diabetes medicine, hypertension medicine.
