# Supplementary material for: Association of serum lycopene concentrations with all-cause and cardiovascular mortality among individuals with chronic kidney disease: A cohort study
Source: Front Nutr. 2022 Dec 5;9:1048884. doi: 10.3389/fnut.2022.1048884 (PMC9760801; doi:10.3389/fnut.2022.1048884)
Supplement: Supplementary file 2 [file Table_2.docx]

**Supplementary Table 2 Subgroup analyses of the associations between serum lycopene concentrations and CVD mortality among patient with CKD stage 3-5**

|  | **Serum lycopene concentration(μg/dl)** | | | | | | | | | | |  |
| --- | --- | --- | --- | --- | --- | --- | --- | --- | --- | --- | --- | --- |
| **Characteristics** | **Quartile 1** | | **Quartile 2** | | **P** | **Quartile 3** | | **p** | **Quartile 4** | | **p** | **P _interaction_** |
|  | **No. deaths/total** | **Reference** | **No. deaths/total** | **HR (95%CI)** |  | **No. deaths/total** | **HR (95%CI)** |  | **No. deaths/total** | **HR (95%CI)** |  |  |
| Sex |  |  |  |  |  |  |  |  |  |  |  | 0.8375 |
| female | 337/911 | 1 | 299/900 | 0.91 (0.776-1.068) | 0.2477 | 207/727 | 0.862 (0.722-1.029) | 0.0999 | 155/604 | 0.794 (0.645-0.976) | 0.0288 |  |
| male | 250/648 | 1 | 184/517 | 0.966 (0.793-1.176) | 0.729 | 169/533 | 0.815 (0.663-1.002) | 0.0523 | 119/447 | 0.735 (0.575-0.939) | 0.0138 |  |
| Age, year |  |  |  |  |  |  |  |  |  |  |  | 0.731 |
| ＜65 | 70/340 | 1 | 79/447 | 0.973 (0.698-1.356) | 0.8718 | 66/470 | 0.7 (0.494-0.991) | 0.0446 | 56/399 | 0.744 (0.509-1.088) | 0.127 |  |
| ≥65 | 517/1219 | 1 | 404/970 | 0.811 (0.711-0.926) | 0.002 | 310/790 | 0.702 (0.608-0.811) | <0.001 | 218/652 | 0.588 (0.496-0.698) | <0.001 |  |
| Race/ethnicity |  |  |  |  |  |  |  |  |  |  |  |  |
| Non-Hispanic White | 366/888 | 1 | 327/863 | 0.955 (0.819-1.112) | 0.5501 | 251/741 | 0.868 (0.735-1.025) | 0.0958 | 190/667 | 0.764 (0.631-0.927) | 0.0062 |  |
| Other | 221/671 | 1 | 156/554 | 0.904 (0.732-1.116) | 0.3493 | 125/519 | 0.823 (0.655-1.035) | 0.0952 | 84/384 | 0.854 (0.648-1.125) | 0.2619 |  |
| BMI, kg/m^2^ |  |  |  |  |  |  |  |  |  |  |  | 0.019 |
| ≥30 | 162/423 | 1 | 142/392 | 1.063 (0.842-1.342) | 0.6085 | 110/399 | 0.816 (0.633-1.05) | 0.1144 | 82/333 | 0.749 (0.556-1.011) | 0.0586 |  |
| ＜30 | 425/1136 | 1 | 341/1025 | 0.896 (0.774-1.037) | 0.1407 | 266/861 | 0.874 (0.745-1.024) | 0.096 | 192/718 | 0.802 (0.665-0.966) | 0.0203 |  |
| Serum triglycerides, mg/dl |  |  |  |  |  |  |  |  |  |  |  | 0.241 |
| ≥200 | 133/337 | 1 | 116/348 | 0.868 (0.671-1.124) | 0.2834 | 104/297 | 0.98 (0.75-1.282) | 0.8846 | 82/281 | 0.845 (0.623-1.146) | 0.2786 |  |
| ＜200 | 454/1222 | 1 | 367/1069 | 0.951 (0.826-1.094) | 0.4814 | 272/963 | 0.809 (0.693-0.945) | 0.0076 | 192/770 | 0.762 (0.633-0.916) | 0.0038 |  |
| Serum total cholesterol, mg/dl |  |  |  |  |  |  |  |  |  |  |  | 0.538 |
| ≥240 | 165/423 | 1 | 188/468 | 0.932 (0.752-1.154) | 0.5176 | 158/472 | 0.788 (0.631-0.985) | 0.0363 | 165/549 | 0.758 (0.602-0.953) | 0.0179 |  |
| ＜240 | 422/1136 | 1 | 295/949 | 0.942 (0.808-1.097) | 0.4409 | 218/788 | 0.879 (0.742-1.042) | 0.1378 |  |  |  |  |
| Diabetes |  |  |  |  |  |  |  |  |  |  |  | 0.523 |
| yes | 117/293 | 1 | 111/262 | 0.922 (0.704-1.207) | 0.5542 | 69/214 | 0.79 (0.581-1.075) | 0.1333 | 67/228 | 0.693 (0.496-0.967) | 0.0308 |  |
| No | 470/1266 | 1 | 372/1155 | 0.972 (0.845-1.117) | 0.6875 | 307/1046 | 0.875 (0.753-1.016) | 0.0788 | 207/823 | 0.831 (0.695-0.995) | 0.0436 |  |
| Hypertension |  |  |  |  |  |  |  |  |  |  |  | 0.871 |
| yes | 356/847 | 1 | 310/783 | 0.955 (0.817-1.117) | 0.5645 | 240/680 | 0.825 (0.697-0.977) | 0.0257 | 198/660 | 0.748 (0.618-0.905) | 0.0029 |  |
| no | 231/712 | 1 | 173/634 | 0.923 (0.753-1.131) | 0.4397 | 136/580 | 0.924 (0.738-1.157) | 0.4897 | 76/391 | 0.899 (0.681-1.187) | 0.4533 |  |
| Current smoking status |  |  |  |  |  |  |  |  |  |  |  | 0.464 |
| yes | 231/712 | 1 | 173/634 | 0.755 (0.521-1.095) | 0.1386 | 136/580 | 0.809 (0.563-1.163) | 0.2525 | 76/391 | 0.518 (0.3-0.892) | 0.0177 |  |
| no | 501/1321 | 1 | 436/1243 | 0.952 (0.835-1.086) | 0.4636 | 322/1081 | 0.836 (0.724-0.966) | 0.015 | 253/934 | 0.769 (0.654-0.905) | 0.0016 |  |
| Current drinking status |  |  |  |  |  |  |  |  |  |  |  | 0.723 |
| yes | 73/228 | 1 | 66/198 | 0.96 (0.68-1.355) | 0.8155 | 57/183 | 1.03 (0.724-1.466) | 0.8674 | 29/128 | 1.049 (0.67-1.644) | 0.8345 |  |
| no | 514/1331 | 1 | 417/1219 | 0.896 (0.785-1.023) | 0.1034 | 319/1077 | 0.801 (0.693-0.926) | 0.0027 | 245/923 | 0.706 (0.599-0.832) | <0.001 |  |

Data are presented as HR (95% CI). Adjusted for age (continuous) ，sex (male or female)，race/ethnicity, education level, poverty to income ratio, BMI, uric acid, triglycerides, total cholesterol, smoking status,

drinking status, diabetes, hypertension, diabetes medicine, hypertension medicine.
