# Supplementary material for: Association of serum lycopene concentrations with all-cause and cardiovascular mortality among individuals with chronic kidney disease: A cohort study
Source: Front Nutr. 2022 Dec 5;9:1048884. doi: 10.3389/fnut.2022.1048884 (PMC9760801; doi:10.3389/fnut.2022.1048884)
Supplement: Supplementary file 3 [file Table_3.docx]

**Supplementary Table 3 Results of sensitivity analyses of the associations between serum lycopene concentrations and mortality**

| **Quartiles of serum lycopene level** | **All-cause mortality** | | | **CVD mortality** | | |
| --- | --- | --- | --- | --- | --- | --- |
|  | **Event/total** | **HR (95%CI)** | **P value** | **Event/total** | **HR (95%CI)** | **P value** |
| **Excluding those died within the first follow-up 12 months** | | | | | | |
| Quartiles 1 | 1593/1878 | Reference |  | 682/1878 | Reference |  |
| Quartiles 2 | 1343/1830 | 0.911 (0.846-0.982) | 0.0142 | 543/1830 | 0.904 (0.805-1.014) | 0.0843 |
| Quartiles 3 | 1115/1876 | 0.802 (0.741-0.868) | <0.001 | 458/1876 | 0.828 (0.732-0.936) | 0.0026 |
| Quartiles 4 | 963/1887 | 0.79 (0.724-0.863) | <0.001 | 377/1887 | 0.799 (0.696-0.918) | 0.0015 |
| **Using data before multiple impution** | | | | | | |
| Quartiles 1 | 1059/1229 | Reference |  | 448/1229 | Reference |  |
| Quartiles 2 | 814/1111 | 0.876 (0.798-0.962) | 0.0056 | 343/1111 | 0.901 (0.78-1.041) | 0.1579 |
| Quartiles 3 | 636/1058 | 0.788 (0.711-0.873) | <0.001 | 272/1058 | 0.84 (0.718-0.983) | 0.0298 |
| Quartiles 4 | 428/888 | 0.757 (0.669-0.856) | <0.001 | 179/888 | 0.794 (0.657-0.96) | 0.0174 |

Data are presented as HR (95% CI). Adjusted for age (continuous) ，sex (male or female) race/ethnicity, education level, poverty to income ratio, BMI, serum uric acid, triglycerides, total cholesterol, smoking status, drinking status, diabetes, hypertension, diabetes medicine, hypertension medicine.
